# A Lecture on the Modern Improvements of Surgery

**Published:** 1845-07

**Authors:** John Houston


### SELECTIONS

FROM

### AMERICAN AND FOREIGN JOURNALS.

*A Lecture on the Modern Improvements in Surgery.*—(Delivered 4th November, 1844.) Being Introductory to a Course of Lectures on Surgery in the School of Medicine, Park-street, Dublin. By John Houston, Esq., M. D. M. R. I. A. Apology—Chemistry and Histology—The blood—Inflammation—Union by the first intention—Military surgery—Amputation—Lower jaw—Joints—Subcutaneous sections—Autoplasty—Aneurism—Nævus—Tracheotomy—Hydrothorax—Tympanitis—Hydrocephalus—Fractures—Artificial anus—Urinary diseases—Lithotomy and Lithotrity—Rectum—Syphilis.

[CONCLUDED.]

Closely connected with this treatment is that to which the term *autoplasty* is applied. This, which in the first instance was almost confined to separations of lost or mutilated noses, is now applied to an innumerable variety of other similar lesions. Taliacotius in the year 1597, wrote a book on the separations of noses, and although, for long after the appearance of his work, his statements were not credited beyond his own locality, there can be no doubt now, from recent experience of the practice, of his having succeeded well in such operations. His fellow citizens of Bologna certainly believed in his surgical skill in this way, and also venerated his private character, as they erected to his memory a monument, representing him as clad in his professional toga, and holding a nose in his right hand.

From the time of Taliacotius, for nearly 300 years, we hear little of the operation. Perhaps satire may have had to do with the disrepute into which it fell. A few stanzas like the following, from Hudibras, were well calculated to bring it into discredit:—

"So, learned Taliacotius, from
The brawny part of porter's bum
Cut supplemental noses, which
Would last as long as parent breach;
But, when the date of Nock was out,
Off dropt the sympathetic snout."

In the year 1814, Mr. Carpue, of London, re-introduced it, and from this date up to the present time, the number and variety of the applications of the principle have become so great, that *autoplasty* has been reduced to a science, with names for the different classes and orders of the departments. Thus we have in connexion with the face alone, the operations of *cheiloplasty* (restoration of lips); *stomatoplasty* (opening up of a closed mouth); *rhinoplasty* (cementing deformities of noses); *genoplasty* (the art of restoring cheeks); *blepharoplasty* (repairing eyelids); *keratoplasty* (supplying a new cornea for the eye); *otoplasty* (mending the pavilion of the ear), &c.

There are three different principles or modes on which these operations are conducted.

The first, the Italian mode, as it is called, is that by transplantation; as, for example, where deficiency of the nose is supplied by a piece taken from the arm or other part of the body [showing plates].

The second, the Indian method, or that by twisting; as where the dificiency is made up by dissecting a triangular flap of skin off the forehead, and bringing it down on the nose by such a twist as will permit the outside of the skin to retain its forward position on the face.

The third, or French method, the principle of which consists in pushing or sliding a piece of integument from one place and making it grow on another, to fill up some defect in that direction; as where, after the removal of the scar of a burn, the neighboring skin is loosened from the surface underneath, and brought so as to grow over and fill up the gap.

This Book of Beauty [showing M. Serres's work] shows the practice of one gentleman in this way. There is, perhaps, a good deal of picture-making in it; but to those who may be credulous on the matter, I beg to show a few pictures of cases of my own, for the accuracy and freedom from exaggeration of which there are many witnesses, and which tempt me to concur in the expression uttered by M. Roux —"Qu'il n'y avait rien d'impossible en faire des restaurations de la face."

The operation of palato-raphé is one of the most interesting and beautiful applications of autoplasty. M. Roux, to whom we are indebted for so much light regarding it, has operated upwards of one hundred times in patients of all ages. I was myself one of the first in this city to practice it successfully [plates]. Sir P. Crampton, Mr. Cusack, and Mr. Hamilton, have also been successful in similar cases. Here is Roux's first instrument for palato-raphé; and here is one of great ingenuity and efficacy, lately invented by Mr. Samuel M'Clean, of this city.

The extension of the practice of autoplasty to other regions of the body has been highly successful. Thus, the canal of the urethra has been restored by Earle, Cooper, and Dieffenbach, by pieces of skin taken from the scrotum. Jobert has applied autoplasty to the cure of vesico-vaginal fistula; Jameson, of Baltimore, and Gerdy, to the radical cure of hernia. Mr. Syme has, I am informed, cured fungi of the testis, which have been first proved by the microscope not to be cancerous, by covering them over with flaps of the integuments, brought from the sides of the tumor. And Clot Bey has successfully practised orcheoplasty even at Alexandria in Egypt; all which I mention as showing how the science of autoplasty, scarcely known more than thirty years, is already cultivated in numerous parts of the world.

Then there is the disease of *aneurism* [pointing to a drawing of a popliteal aneurism, which had caused death by inward bursting and extravasation among the muscles], that terrible malady in which the person who has the misfortune to labor under it—doomed to a premature and sudden death—looks with awful anxiety and trepidation at the throbbings of his heart in the tumor, not knowing how soon the moment may arrive when, by its bursting, his life's blood shall gush out and hurry him into eternity. This disease, for the cure of which ingenuity was supposed to have been exhausted, has within the last year, and, I am proud to say, in this city, been the subject of a new and brilliant light. The hitherto ordinary and most approved method of cure in this affection has been that of tying the artery leading to the tumor, so as to arrest the current of blood and cause it to coagulate in the artery. But this method has one serious drawback—namely, that it involves the performance of a surgical operation, in itself a source of danger to life. The improvement I allude to, and which has been yet only practised for aneurism of the extremities, particularly popliteal aneurism, consists in accomplishing the same cure without performing any operation at all. The knife and ligature, with all their risks and terrors, are here laid aside, and simple pressure on the main artery leading to the tumor substituted in their stead. To my esteemed friend, Mr. Edward Hutton, is due the introduction into practice of this improvement, one which places his name beside, if not before, those of Hunter and Hodgson, as a benefactor of mankind in this department of surgery.

I find that a case of popliteal aneurism was published in 1831, by Assalini, as having been cured by him by means of pressure on the femoral artery; but his practice has been little heard of and less copied. The late Professor Todd, too, of this city had also, it is true, attempted a similar mode of treatment; but as neither of those cases were known to Mr. Hutton at the date of his first successful experiment, the method adopted and advocated by him may, truly, be regarded as original in his hands.

I have witnessed the development of this improved mode by practice with great interest and satisfaction, and, as far as trials have yet gone, it appears to be not only infinitely more safe than the old plan, but even more efficacious, as out of seven cases of popliteal aneurism, treated in this way in Dublin, within the short period of one year, there has been only one failure, and in that the fault lay in the patient and not in the method of treatment. Following the example set by Mr. Hutton—Dr. Cusack, Dr. Bellingham, Dr. Kirby, and Dr. Harrison have treated cases in this way, and all with equal success. Dr. Bellingham's case, in which a second popliteal aneurism, in the same individual was cured by this means, appears to me of particular value, as doubly proving the efficacy of the remedy. Within the last fortnight, a gentleman of great consequence and of large fortune, but with a constitution under which no operation could have been practised without imminent risk to his life, has been by this simple means, in the hands of Dr. Cusack, cured of a popliteal aneurism of such a size as to have already, in part, made a bursting. The practice has been also tried and approved of in London, by Mr. Liston. I regard it as decidedly one of the most important improvements of the day, in this or in any other country—not inferior even to lithotrity in its capability of effecting positive good, and certainly far its superior in the negative quality of being incapable of doing harm. [Here Dr. Houston exhibited the instruments used for compressing the artery, showing the several improvements which they have undergone from that first employed by Mr. Hutton to that last used by Dr. Cusack.]

*Nævus*, or *Aneurism by Anastomosis*, is another affection of the bloodvessels; in the treatment there has been great improvement of late years. These disfiguring and dangerous marks were formerly, when meddled with at all, treated by excision; and the directions most strongly inculcated were to the effect that the act of removal should be accomplished with as much expedition as possible, in order that danger might not arise from the profusion of hemorrhage which was known always to attend the operation. But danger to life was not the only inconvenience of this mode of remedy. Great disfigurement necessarily follows it, when, as frequently happens, some part of the face, such as the eyelids, the nose, or the lip [plates], happens to be the seat of the disease. A more accurate knowledge of the nature of the affection has led modern surgeons to the practice of curing it by the production of inflammation in its texture, and this they effect in a variety of ways, as, by the introduction of setons, sulphate of copper, nitrate of silver, or other irritating material, into its substance; or else, by acting upon it from without, through the medium of stimulating washes, or by inoculation with the vaccine virus. The inflammation thus produced is first followed by an enlargement and hardening of the part, which gives place in a little time to a diminution of bulk, and a return to a somewhat normal state. The orator Cicero derived his name from a pealike nævus of this kind on his nose (*cicer arietinum*, vetches); and if the surgeons of his day had been skilled in the simple means now in practice, and had applied it in his especial instance, doubtless—not to speak of the satisfaction which such a riddance would have been to the gentleman himself—we, at all events, should have been made acquainted with him by some other cognomen.

On the subject of *Tracheotomy*, a very remarkable incident has recently occurred, suggesting new views regarding the performance of that operation. The case of the celebrated engineer, Mr. Brunel, in which half-a-sovereign entered by accident, and remained in the windpipe, has been heard of by all the world. The mechanical ingenuity of Mr. Brunel suggestad to himself the plan of inverting his body so as to allow the coin to run out of, as it had run into, his patulous trachea, and he accordingly made the attempt; but there was an element, a principle here to be encountered, such as is never taken into calculation in estimating the facilities and difficulties involved in the construction of locomotive or atmospheric engines, and which the medical man alone is competent to deal with—namely, a self-acting spasm, the effect and the attribute of vitality, resisting the passage of any foreign irritating substance. Mr. Brunel's plan, in itself, therefore, failed, and the practical cause of its failure lay in the violence of the spasm which the coin produced in the muscles of the rima glottidis at the moment of its contact with that opening; indeed, he was nearly suffocated in the attempt. And here it was that the knowledge of the surgeon, superadded to that of the mechanist, completed the triumph of genius and of art, and saved the man. Sir Benjamin Brodie made an opening into the windpipe close behind the point at which the spasmodic action was known to occur, partly with a view of quieting the spasm (an effect, by the way, first pointed out by my friend Professor Porter, as one of the results of tracheotomy) but chiefly to give air to the lungs by a new and artificial route,, and thereby afford time and opportunity for the foreign body to get past the obstruction. Mr. Brunel's body was then, as before, inverted, and, as was anticipated, the coin ran without farther obstruction from the lungs into the mouth. Never did artistic and scientific skill combined, produce a more marked, a more happy effect. The whole world rang with applause and congratulation.

There are some of what are considered purely medical subjects, to which surgical assistance has been of late most felicitously applied. One of these is what may be termed *acute hydrothorax*, a rapid and great effusion into the bag of the pleura, and the practice to which I allude is that of tapping the side with a fine trocar, so as to give escape to the fluid. A remarkable instance of this novel and bold practice lately occurred in this city, in the practice of my friend Dr. Stokes. A lady, the subject of this affection, was in great agony, and in danger so imminent that her pulse was gone, and she had passed from delirum to coma. On Dr. Stokes's recommendation, the operation of paracentesis thoracis, in the manner just spoken of, was performed. Immediate relief was given, and the patient got rapidly well. Another instance has been related to me by Dr. Stokes, of the same affection, only more chronic in character. Here, the bag of the pleura was so filled and distended, that the heart was pushed by the fluid from the left into the right side of the chest, and the ribs were driven out in the opposite direction. The same plan of treatment was adopted, and with equal success. Even during the process of drawing off the fluid, which amounted to several gallons, the lung could be followed by the stethoscope rising into its place against the ribs, and the heart could be tracked from right to left, until it had finally settled in its natural position. A remarkable phenomenon attended on the sudden change of place in the parts, even although that change had been from an unnatural to a natural position. The lungs and heart became both so violently agitated by the movement, as almost to have had their functions suspended; but, in a little time, and aided by due medical attentions, they settled again—if I may so speak—to their work, and the patient got completely well. The innovation and improvement, as regards treatment, are here two-fold; first, in operating at all in such cases; and, secondly, in substituting a small trocar for the scalpel—the latter change being of so radical a nature as almost to convert the practice by operation into one without operation.

The same kind of practice has been applied, by Sir Henry Marsh, to excessive *distention of the abdomen from flatus*, with the most soothing result, and without any bad effects from the operation. I knew a lady on whom Mr. Cusack, operated several times in this way, in consultation with Sir Philip Crampton and Sir H. Marsh—the operation being performed with a fine trocar and canula,—and each time with much relief to the patient from the most painful suffering.

Even the cavities of the skull are not beyond reach of the exploring hand of the surgeon. Many cases have been of late recorded, and particularly one by my friend, Mr. Richard Butcher, in which the fluid of *hydrocephalus* has been drawn off by tapping, and, as is stated, often with favorable results.

*Fractures of bones* are not, at the present day, regarded with any of that alarm and apprehension, nor attended with the after inconveniences which formerly succeeded to such accidents. The principles which regulate the growths of new bone, thrown out for the purpose of reparation under injury or disease, are now so well understood, that we comprehend, in every case, as if carried on before our eyes, the several phenomena, in the exact order in which they actually take place, from the beginning to the ending of the process. We know, too, what circumstances are calculated to promote or retard, to render perfect or to spoil, the new growth; and if, from unforeseen or accidental causes, reparation does not go on, we have discovered the means by which to direct it aright, and to conduct it to a perfect consummation. The means to this end I shall explain to you on another occasion. As to the mechanical derangements attendant on fractures, and their remedies, they are now known with almost mathematical precision. Tell me, for example, the part of any given bone broken, and the cause of the accident, and I shall be able to inform you, without seeing the patient, of the precise nature of the displacement which the fragments have undergone, and, of course, to suggest the mechanical appliances fittest for restoring and keeping them to the right place. Certain fractures, such as Colles's of the radius, Pott's fracture near the ankle, &c., having had attention directed to them by being made the subject of special memoirs, are well known, in this light, by the profession: but it is a fact, and a most important one to be borne in mind, that all fractures, as well as these, are respectively equally fixed and determinate in their mechanical characters and consequences. In a fracture of the middle of the thigh, for example, there is rarely an exception to the rule that the lower fragment, rotated outwards on its axis, lies on a plane behind the upper fragment, in this way [showing specimens], giving rise to two marked kinds of deformity—namely, shortening of the limb and turning out of the toes; and any surgical appliances adopted here should of course, have especial reference to the removal of these two defects, and to the prevention of their return during the period of the healing process, otherwise the patient will have been badly cured, and will feel and complain of the effects of the accident while he lives. Thus, if he happens to be fond of the chase, he will never after sit his saddle in comfort or in safety, from the mechanical change which has been allowed to come over his thigh-bone, at the seat of the fracture, converting it, from a bow-like arch which fits it as if intended by nature for horse-riding, into a straight line, or even changing the convexity of the bow from the outer to the inner side, in a manner to incapacitate it completely for such a purpose. Or, if he be a pedestrian, his limping gait and everted toe will remind him, every morning he rises, of his double misfortune; first, in having met with a bad accident, and secondly, with a bad surgeon—one, as he might say, unacquainted with the modern improvements of his art.

Our knowledge, at present, of the different conditions and results of fractures of the *neck of the thigh-bone*—an accident so common and so afflicting in old age—stands in striking contrast with that of our immediate predecessors. It was held by them that such fractures never became re-united by bone, and, as a consequence, attempts at such a mode of cure were discountenanced as not only unnecessary, but injurious. Such was the theory and practice of even the great Sir Astly Cooper. But how far is this changed? We now know that such cases are not only possible, but common. I saw this day an old gentleman who, about two years and a half ago, was under my care for complete, but impacted fracture of the neck of the the thigh-bone. There is now perfect consolidation of the fracture, and he walks steadily and well without the aid of either crutch or stick. We know, too, at sight, what cases admit of union, with tolerable certainty, from those in which the chances of such a happy result are few; and, still further, what is of equal consequence, we have learned the important fact, which all should know, that one of the conditions of this fracture, on which depends mainly the chance of reparation—viz., a certain remaining degree of coaptation, and the presence of certain unbroken shreds of the softer tissues—is such as to forbid a practice justifiable, or even necessary for diagnosis, in other fractures—namely, that of seeking for what is termed crepitus, inasmuch as the act of doing so may displace the fragments and tear asunder the only remaining elements of future union, thereby taking away all chance of reparation. Want of due attention to this point may have been one of the causes of such infrequency of union in former times. Here, again, justice and candor compel me to introduce the name of certain of my countrymen; for it is a fact, that to Dublin is mainly due our knowledge of these important facts regarding fractures of the neck of the thigh bone, as the writings of the late much-honored Professor Colles, and of my friends, Dr. Adams and Dr. Smith, bear evidence. Besides, our museums abound in reunited fractures of this kind; some of them without, others within, the capsule: sufficient, truly, to justify the promulgation of the principles and practice to which I have adverted, and to serve at once as the evidence and the basis of a new fact in surgery.

Some most important considerations have lately been brought before the profession by M. Amussat, of Paris, in reference to the establishment, by operation, of an *artificial anus* in certain cases of total obstruction of the bowels. The operation consists in making an opening through the abdominal parietes into the ascending or descending colon: and the cases in which it may be required are—first, some extreme varieties of imperforate anus in infants; and secondly, cases of invincible constipation from mechanical obstruction of the great intestine. Obstructions of a non-malignant nature, involving danger to life, may, by a counter-opening of this kind, letting loose the pent-up excretions, have their fatal tendencies counteracted; and, even in cancerous obstructions of the bowel, a new anus may, in the same way, soothe pain and prolong life. Such an operation might, in cases of this nature, be sometimes undertaken with as much reason, and in promise of as much good as some which are performed for strangulated abdominal hernia. Forty-three operations of this kind are recorded as having been performed: eighteen on adults, and twenty-five on infants, and out of these, I may state, as showing at least that the operation is practicable, and to a certain extent safe, that one patient, a girl, two days old, operated on for artificial anus, by making an opening in the sigmoid flexure of the colon, was alive and well at nineteen years of age; another, a boy, two days old, operated on in the same manner, and in the same part, lived to between sixty and seventy years of age; and a third, a woman, in whom the operation was performed on the descending colon at the age of forty-four, was alive seventeen years subsequently. The history of the operation is very interesting. About 1710, M. Littré recommended the opening of the sigmoid flexure of the colon for imperforate anus. In 1776, M. Pillore made an artificial opening into the cæcum, in an adult whose rectum was obstructed by a cancerous tumor. And in 1793, the individual whom I have above alluded to as having lived to old age, was operated on by MM, Duret, Dubois, Dessault, and Dupuytren, and others have tried the operation with various degrees of success, but it is to M. Amussat—who has investigated the subject in a scientific manner, and tested the practicability and value of it by at least ten operations, as well on adults as on children—that we are indebted for the encouraging prospects which it at present holds out. The alternative of an artificial anus—an opening, in an unwonted and inconvenient situation, for the discharge of the excrement, is indeed a sad one, but, such as it is, it stands as one of the many illustrations of the increasing power of our art to smooth the pillow of sickness, or even to avert the stroke of death, when all other earthly aids have failed.

There is no operation, either in ancient or modern surgery, to be compared with that for the removal of *diseased ovaria*—an operation of very recent introduction, and one which, when the extent and depth of the incisions, and the importance of the parts exposed in it are considered, makes one shudder at the mere thought of it. Think, for a moment, of an operation in which the abdomen of a female, from top to bottom is laid open by the knife—a great tumor, perhaps the size of the head, turned out for removal, and the delicate vital organs of the cavity exposed to the unwonted air, or the intrusive hand; think of all this, and then you can form some estimate of the operation termed *ovariotomy*. Yet, strange to say, this operation has been performed seventy-four times; and, stranger still, nearly one half of the patients have recovered. The question is yet to be decided, whether such an operation is at all justifiable—viz., whether, on the whole, more good or evil is likely to follow its introduction into general practice. But the grand question is settled—we have the facts before us—that the human frame is abundantly capable of sustaining, and even of recovering perfectly from the effects of it. The great impediment to the settlement of the question of propriety is, the difficulty of determining, beforehand, the fit, from the unfit cases: of determining those in which the tumor is insulated and movable in the cavity, from those in which it has contracted adhesions to the parts around: as in the former, the tumor admits of being turned out with facility, and rapid convalescence follows the operation: whereas in the latter, the surgeon, afrer having opened the abdomen, is obliged to desist in the midst of a terrible operation, leaving the unfortunate patient sadly the worse for his interference.

Diagnosis, then, is here the great desideratum. When our tact, in this respect, becomes so perfect that we can single out those cases which are not adherent, inside, from those which are so; and, still farther, when there shall cease to be any grounds of fallacy as regards the presence or absence of such a tumor—for it is a strange fact, that the operation of ovariotomy has been more than once performed when there was no tumor at all to be found, upon the abdomen being laid open—when those points are settled, then, indeed, may this operation take a place, not only among the most remarkable, but also among the most valuable of the modern improvements in surgery,

In the wide field embraced under the term *urinary diseases*, most striking and important improvements have been, of late, effected; perhaps to no other class has there been so felicitious an application of the twin sciences of chemistry and histology.

The due discharge of the functions of the kidneys is essential to the general well-being of the frame. The slightest inefficiency in this respect is felt injuriously somewhere, while serious lesion is fatal. And, on the other hand, changes in the health at large invariably disturb, in a palpable manner, the functions of the kidneys. These organs are then. truly, the barometers of the health, the urine representing, with the greatest delicacy, the quicksilver—only requiring a chemical and microscopic eye for the detection of its degrees and kinds of pathological changes.

The examples of derangements of the kidney producing general derangement of health, are numerous, and in all such it is chiefly by an examination of the urine that the nature of the lesion is to be ascertained. This is wherein the novelty lies. Persons have been long and lingeringly ill, and incapacitated from the ordinary duties of life. They have had dropsies, shiverings, convulsions, or even madness; and sometimes all these ailments together. They have been attended by the most skilful physicians, and they have died. Nevertheless, neither during lifetime, nor *post-mortem*, was the nature of their malady known. They have been reported as having died of cerebral or liver disease, or simply of dropsy, & no doubt they were treated during life with a view to the cure of such suppositious affections. Hundreds, in all ages, have pined away thus; and yet it is only within the last ten or fifteen years that a full and satisfactory solution of the riddle has been made out. It was reserved for the genius and perseverance of Bright to trace these protracted conditions of illness, and those unstayed and melancholy deaths to their true source—viz., slow, insidious, and oftวn almost unappreciable degeneration of the kidneys, now well known as the *morbus Brightii.* This disease, although in itself but little striking—and therefore so long passed over unobserved—is, nevertheless, sufficiently decided to spoil the secretion of the kidney, and send one of its most noxious excretions—the urea—which should be discharged with the urine, back through the frame, poisoning the springs of life at their source, and thereby agitating or paralyzing every vital function.

The pathological condition of the kidney, characteristic of such cases, is now well known and established; but what is the evidence during life of its presence? What is the outward test of its existence at that period when a knowledge of it is most beneficial—viz, while it is still curable? The test is a chemical one—namely, the precipitation of albumen from the urine by nitric acid or by heat; and on this fact, simple as it may appear, so much stress is now laid, that a person with a permanently albuminous state of his urine is scarcely considered an eligible life for insurance, and, still more, such a person, it is well known, has a bad chance of recovery, if unfortunately he should happen to become the subject of any severe accident or operation.

There are various deposits or sediments found in the urine, the results and the evidences of certain corresponding diseases, and the exact nature of which—often a matter of great moment in diagnosis—can only be determined by the aid of the *microscope.* Such are globules of blood, of pus, or of mucus, or more especially crystals of salts, every variety of which is charactered by a special and definite form [showing a diagram], and the ascertaining of which determines with certainty the nature of the disease present. A case which I met with lately will illustrate this point. A medical gentleman asked my advice under the followiug circumstances:—He had gone to bed the night before in his ordinary health, but awoke in the middle of the night with excruciating pain in his right side, deep in the abdomen, and shooting down into the thigh. The pain had lasted two or three hours, and then subsided, leaving a sense of numbness in its place. He had little other complaint to offer. Suspecting from the symptoms the presence of a stone in the kidney or ureter, I subjected the urine to a microscopic examination, when two different objects, each of great value towards the diagnosis, presented themselves to my view—one scattered globules of blood, the other crystals of the oxalate of lime, those beautiful octahedral crystals discovered lately by Dr. Golding Bird. The latter I regarded as evidences of the oxalic acid diathesis, and of the existence of a stone somewhere; the former I looked upon as proving that stone to have been in motion, and in its transit from one place to another, to have torn the surface of the lining membrane. Such were my inferences from those microscopic data; and on them I founded the opinion which I gave, and the treatment which I recommended. I said to my patient, that perhaps, without any further pain, but most likely, after a few more such stings as he had before experienced, he would probably void a small stone, which, in such event, would certainly be of the oxalate of lime or mulberry kind. Of course I directed what I considered appropriate treatment in the meantime. On the fifth day thereafter, this gentleman again called upon me. He told me, that on the third night succeeding to that on which he had been first unwell, he experienced a similar violent attack, but that it went off rather suddenly and unexpectedly, when he became entirety free from pain. He handed me this calculus [showing one,] which he said he had passed with his water the morning after. It is a true mulberry, and of such a roughened and spiculated charac- r. that blood (and it was that which the microscope detected) must have been inevitably drawn, by its forced passage along the narrow tube of the ureter. Here, the microscope not only ascertained the actual presence of a stone, but by determining its nature and composition indicated the treatment applicable to the removal of the diathesis which generated it, and the consequent prevention of the growth of such another—an accuracy and value of diagnosis unattainable, I believe by any other known means.

*Lithotomy*, invented for the relief of pains more cruel, according to the expression of Lomius, than any left for mankind in the box of Pandora, is one of those operations the longest known. Æsculapius practised it; but Hippocrates made his disciples swear that they would not perform it, no one knows why, but most probably because he thought it too difficult for them. Physicians say because he was a physician. By the way, I may be here permitted to observe, touching the relative rank of surgery and physic, that—as far, at least, as regards primogeniture—the latter must give way to the former, as Æsculapius, the God of Surgery, lived upwards of seven hundred years before the time of Hippocrates, the acknowledged deity of medicine. The history of this operation, of the different modes of performing it, of the innumerable instruments invented for it, and of the rivalry among its practitioners in different ages and countries, is exceedingly curious and interesting, but is a subject unsuited for the present occasion.

*Lithotrity*, on the other hand, is an operation so completely of our own days, that few of the latest dictionaries in surgery do more than mention its name. It has sprung, phœnix-like, into perfection, and stands now forth triumphant over disease. The operation of lithotomy has always, hitherto, ranked as the chef d'œuvre of surgery; lithotrity, where it suits, takes from it the palm. A monk of the name of Citeau, and a Major Martin, broke down calculi in their own bladders—the first, by means of a tube, or canula, and a steel rod, on which he struck with a hammer, as a statuary strikes his chisel; the second, by means of a file, introduced likewise through a canula; and the stones thus broken down were discharged, piecemeal, with the urine. These two operations, performed long ago, at the dictation of painful suffering and ingenuity, are the prototypes of the *hammer* of Heurteloup, and the drill of Civiale, the inventors and the practitioners of which are still the living ornaments of this age of improvement. The talents of my friend, Mr. Francis L'Estrange, displayed themselves in some ingenious and highly important improvements in the brisépierre, or crushing lithotrite. [Here Dr. Houston exhibited several of the most remarkable instruments, both for lithotomy and lithotrity, and also a very ingenious one, which he said was invented by Leonard Trant, Esq., for removal of calculi from the urethra.]

While speaking of the services rendered to surgery by others, I may be permitted to mention at least one or two of those which it has been my own good fortune to contribute. The term *intestinum rectum*, as applied to the lower bowel, I have shown to be a misnomer, leading to incorrect notions regarding its form, and giving rise to most serious errors both in the diagnosis and treatment of some of its diseases. I have demonstrated, in a manner to have satisfied the best anatomists, that the cavity of the rectum, so far from being straight, is formed into several lodges, by projections from its inner walls of very considerable breadth and thickness. These projections I have proved to be of such a nature, as not only to obstruct the passage of bougies introduced with a view of sounding the canal, but to be themselves liable to be mistaken for strictures of the bowel. [For a plate of them, see 'Dublin Hospital Reports' vol. 5.] And this is no supposititious case, for in what other light than a mistake can we regard the printed statements of one of the most approved authors on diseases of the rectum, when he gravely tells us that "more than once has he known the mother and daughters, the father and sons, to be subject to this disease," the fact all the while being that stricture of the rectum is in reality, so rare an affection, that not more than half a dozen examples of it can be found in all the museums in Dublin; and that such a catastrophe as that of thrusting the rude bougie through one of these valves, in mistake for stricture, has in reality occurred, I could, if necessary, give several melancholy proofs. But I believe that such malpractices are greatly on the wane; as an exposition of the true form and disposition of the rectum has not only led to greater accuracy of diagnosis on the part of the qualified practitioner, but has cowed down the empiric, and taken away his best excuse for persistence in his half-ignorant, half-knavish impositions.

As a remedy for internal bleeding hemorrhoids (*vascular tumors of the rectum*], I believe that in recommending the application of the strong *nitric acid* I have rendered a service of no mean amount. Several cases treated in this way may be found in the *Dublin Medical Journal*, and I have permisson from Sir Philip Crampton to give publicity to another such, regarding which he has politely sent me the following note;—

"Dear Houston,—You will be pleased to hear that I operated, about a fortnight since, on Mrs. ——, for the bleeding raspberry excrescence of the rectum, exactly in the manner recommended by you, and that she is now quite well. The disease was of long standing, and her constitution was much reduced by excessive losses of blood. There were three excrescences, each about the size of the thumb-nail; and as they were but slightly raised above the level of the mucous membrane, I considered them to be far better subjects for your opration than for the ligature.

"Faithfully yours,

"Philip Crampton.

"Nov. 12, 1844.

The improvements effected of late years in the diagnosis and treatment of *syphilitic diseases*, are most important and satisfactory. Syphilis, when first introduced into Europe, three hundred and fifty years ago, raged like a pestilence. The world was paralyzed with terror at the mortality which attended it, as, when once it got into a family, the innocent equally with the guilty were in danger of becoming its victims. So great was the terror with which the Parisians regarded the disease, that, by a decree of parliament, dated the 6th of March, 1497, all persons infected with it were ordered, if strangers, to quit the city; if possessed of a home, to shut themselves, up there, so as to be seen by no one; or if citizens, without any fixed place of abode, to repair to St. Germain des-près, where lazar houses were provided for them, and that within twenty-four hours from the date of the order, under penalty of *death*. In the month of September, in the same year, a proclamation was issued by King James the Fourth, in Edinburgh, commanding all persons so affected to repair, before sunset on the next day, to the shore at Leith, where they would find boats provided, ready to transport them to the island of Inch, there to remain until cured; and what was still a harder case, the very surgeons who took charge of such patients were doomed to the same banishment—all equally under penalty of being burned in the cheek with a branding iron. The disease has at the present day lost much of its malignancy, and although some persons die of it such a result is not common. The difference is explained by some on the supposition of the poison being at its highest state of malignancy in its first appearance, and now naturally grown milder. This is probably true in some measure; but it is still more due to the prompt succor afforded to the afflicted, and to the perfection to which the treatment has been brought; for it is a fact, that wherever the science of medicine is most cultivated, there syphilitic diseases are the least virulent.

*Mercury*, the great antidote to the poison of syphilis, has undergone remarkable changes in public estimation. It was not the mere discovery of the antidotal properties of this medicine that effected the good, as mercury in ignorant hands is to this day more likely to be prejudical than serviceable in the treatment of syphilis. But it was in virtue of the results arrived at after a pains-taking observation of its effects, for good or evil, that that accurate knowledge has been arrived at which enables us to employ it with so much potency as a remedy. Paracelsus was the first, in his *Magna Chirurgia*, to recommend the systematic use of mercury internally. But from his day, until comparatively lately, practitioners had no fixed principles regarding it to guide them. Some, and that even within my own memory, judged of its efficacy by the quantity of saliva which it caused to flow out of the mouth in a given time—so many pints a day for example; others, by the amount of medicine taken into the system; some, again, failing to distinguish the ill effects of the remedy from the disease itself pressed the medicine to a most prejudicial and dangerous length, thinking that, in the end, the disorder must give way to its specific influence, while, in fact, one disease was only added to another. Others, not discriminating between affections of a simple and those of a specific nature, have dosed equally with an unsparing hand, as well those who had been better without, as those who stood in need of the mercury; and lastly, others, observing such bad effects from this empirical mode of administering it, and which they did not know how to obviate, abandoned the medicine altogether, and had recourse to vegetable remedies in its stead. Combinations of the ill effects of irregular mercurialization with uncured syphilis stand among the most intractable of diseases, and fortunately under the present improved system of treatment, such are seldom now to be witnessed. Here, however, are a few specimens from our museum, of the cranial bones of some unfortunate patients of bygone days, showing the marks of the *corona veneris*, and of some of the other evils in the form of caries, of which I am speaking. In the museum of the College of Surgeons a number of specimens of this kind are stored up, such as certain future museums will never find matches for. These I have described in detail in my published *catalogue* of that collection.

The great superiority of the surgeon of the present day lies in his skill in distinguishing the true syphilis from simple sores, and in applying to it, and to it alone, the appropriate and necessary remedies. A discrimination between syphilis and simple primary sores was rarely, in former days, attempted. All sores in genital regions were treated with suspicion—*in dubiis suspice luem* being the motto—and terrible injury to the health, and injustice to the character, of individuals was frequently the result. But under our present improved state of knowledge, the well-informed surgeon will never commit such errors. Whenever he determines that mercury is necessary, he knows exactly the time for the administration of the remedy, the symptoms which betoken its salutary effects, the length of time that it should be persevered in, and if it disagrees, how to remedy the evil. He can bring out, in short, every beneficial influence of the medicine on the disease with the utmost exactitude, and at the same time, steer his patient in safety past all its quicksands and dangers. This, I say, is one of the many illustrations which I have to offer of the high state of perfection of modern surgery; and I feel proud in adding, that the labours of our distinguished compatriots, Mr. Carmichael, Mr. Colles, Mr. Hewson, and Mr. Wallace, have largely contributed to such a result. I believe I am safe in saying, that to Mr. Carmichael, in particular, mankind owes a larger debt of gratitude than to any other individual, for the turn which his investigations and writings have given to the treatment of venereal diseases, and for the salutary check put by them to that wholesale and indiscriminate abuse of mercury which disgraced the medical practice of the last century.

Still farther, as regards this subject, a new and additional remedy has been of late discovered, of incalculable value; one which, however, although not, perhaps, like the mercury, a genuine antidote, nevertheless harmonizes and dovetails with that medicine in a most beneficial manner—being of service at the moment when the mercury should be stopped; acting kindly and well, sometimes, when the mercury disagrees; and often, when given at the same time and in combination with that medicine, producing its own good effects while promoting those of the mercury. I allude to the *hydriodate of potash.* Bark, and mineral acids, sarsaparilla, &c., are often given at the same time, and act variously in keeping up and improving the other general health while the poison is being neutralized and eliminated from the system. With these remedies in hand, the well-informed practitioner can, with confidence, promise a safe and satisfactory issue in almost every case. And, after such assurances, if the public continue to place their ailments and their lives in the hands of the mercenary quack—the man to whom all that I have told you, and every thing else on the subject, is unknown—it may truly be said of them, that, in the midst of light, they prefer walking in darkness; that, disregarding the proffered benefits of the accumulated experience of ages, they voluntarily throw themselves back into the condition of danger of those who were banished, by royal ordinances, to desert islands, in company with medical attendants very little better informed than themselves.

A nation's gratitude is due to the medical gentlemen of modern days for the light which their researches have thrown on the subject of *syphilis during intra-uterine life.* It has been discovered that the presence of an occult taint of long-bygone syphilis is prone to be kindled up afresh in the offspring, receiving, as it were, fresh venom, by the new birth, and re-assuming, in the new being, almost all its pristine virulence. Such an infant rarely sees the light of day alive; or, if it do, it is only to propagate to its nurse or others one of the worst forms of syphilis. This is, with a vengeance, visiting the sins of the fathers upon the children. Now, the surgeon has not only traced all this evil to its source, but discovered for it a remedy. And here it may be truly said that his saving hand reaches even to the unborn, staying the course of death, and arresting the spread of evil. A case of this kind, of no uncommon occurrence is the following:—A gentleman marries; he believes himself, and every one else thinks him to be, in the best of health; his virtuous wife is equally so; and every prospect of happiness is open to them. In due time, perhaps prematurely, a child is born, but it is not half thriven; it has the shrivelled skin of old age; it cries continually with a squeaking voice; and is, perhaps, spotted with some eruption. It lingers for a few weeks, or months, pining away daily, instead of adding to its growth, and dies, at last, a miserable object. Well better hopes are entertained for the next time, and the utmost anxiety exists as the period of birth of a second infant approaches, in the hope that *it* may be, unlike the first, perfect and healthy; but such joyous anticipations are again doomed to be blighted, for the infant, when born, worse, perhaps, than its predecesor, may be dead, putrid, and offensive. [Drawings.] Again and again are these disappointed hopes submitted to, and these painful scenes of premature death enacted, the parents themselves all the while continuing in apparent good health. A medical gentleman, properly informed on such matters, is at length, consulted. He questions the father respecting his past history. He finds, on a special inquiry into the point, that the gentleman, at one time had some venereal affection, but long before his marriage, and under such circumstances that he had supposed himself perfectly cured of it. But the acknowledgment is sufficient to confirm his attendant in a well-grounded suspicion that syphilis is at the root of the evil; and full of the confidence which learning and past experience gives, he administers, at the proper time, and with care and judgment, the appropriate remedies to both the parents, who now both require them; after which, the succeeding offspring are delivered into the world, full-grown and healthy—sources of great joy to their parents, and living evidences of the improved state of modern surgery;—I say modern surgery, because the discovery of the connexion of such mortality in new-born infants with syphilis, together with the propper application of the remedy, almost dates within the limits of the last half-century. The "Treatise on Syphilis," by Dr. Colles, contains a chapter on this subject, full of observations of high import and originality.

Gentlemen,—time will not permit me to speak of several other facts which it was my intention to have introduced to your notice—especially some brought to light by the microscope, and which promise to be of great value in practice; such, for example, as the curious and interesting discovery of Mr. Liston as to the presence of spermatozoa in the fluid of some hydroceles: that of M. Gruby, and Dr. Hughes Bennett, regarding the vegetable parasitical nature of certain cutaneous eruptions; and, above all, the novel and important lights shed on the pathology of cancer by the histological researches of Valentin, Gluge, and Müller. I trust however, that what I have said may be sufficient for the object aimed at in this lecture—namely, that of apprising you of the extent, importance, and difficulty of the profession in which you are embarked, and of inciting you to the necessary energy in its prosecution.—*London Lancet.*

